# Severe Shewanella Cellulitis Following Saltwater Exposure in an Elderly Patient

**DOI:** 10.7759/cureus.55724

**Published:** 2024-03-07

**Authors:** Devaun M Reid, Monica Khadka, Sunny Kahlon, Nishanth Chalasani, Kathryn Kass

**Affiliations:** 1 Internal Medicine, University of South Florida Morsani College of Medicine, Tampa, USA; 2 Infectious Disease, University of South Florida, Tampa, USA

**Keywords:** saltwater exposure, sepsis-causing organism, emerging antibiotic resistance, infectious cellulitis, shewanella alga

## Abstract

Shewanella algae is an opportunistic Gram-negative bacillus primarily found in marine environments. It can cause a range of infections in humans, from superficial soft tissue infections to more severe conditions like bacteremia, otitis, and hepatobiliary infections. While infections are rare, they can be significant, leading to complications such as sepsis and tissue necrosis. We present the case of severe cellulitis caused by Shewanella in an 88-year-old patient with multiple comorbidities. Following a blue crab pinch and consequent saltwater exposure, the patient developed severe cellulitis, sepsis, delirium, and atrial fibrillation. Despite these complications and the patient's age, a prompt diagnosis and a combination of antibiotic treatments led to a successful recovery. This case is notable for its illustration of the potential severity and diverse clinical presentation of Shewanella infections. It highlights the importance of considering Shewanella as a possible pathogen in cases of saltwater exposure and teaches management in elderly, multi-morbid patients.

## Introduction

Shewanella algae, a Gram-negative bacillus traditionally found in marine settings, is increasingly recognized as a versatile and opportunistic pathogen. This microbe's ability to inflict a wide array of human infections-from superficial soft tissue afflictions to more critical conditions like bacteremia, otitis, and hepatobiliary infections-highlights its clinical relevance [1]. Despite their relative rarity, the infections they cause are noteworthy for their potential to escalate into severe complications, including sepsis and tissue necrosis [1]. This is starkly illustrated in the case of an 88-year-old patient, who, after a seemingly minor blue crab pinch and subsequent exposure to saltwater, developed severe cellulitis. The incident rapidly evolved into a grave infection marked by cellulitis, sepsis, delirium, and atrial fibrillation, thereby showcasing the pathogen's virulence and its capacity for diverse and severe clinical manifestations, which notably depart from the generally milder symptoms traditionally linked with Shewanella infections.

Shewanella algae's status as a significant emerging pathogen is further substantiated by the surge in reported cases across various geographic regions, including temperate zones such as Portugal [1]. A regional hospital on Lisbon's periphery documented four such instances over a decade, revealing the pathogen's expanding territorial presence. The array of risk factors in these patients - encompassing HIV infection, chronic venous insufficiency, and other comorbidities - underscores the bacterium's predilection for individuals with compromised health [1]. Particularly poignant is the case of a patient whose S. algae bacteremia escalated to septic shock and disseminated intravascular coagulation, underscoring the infection's potential severity [1].

The antibiotic resistance profile of Shewanella, including its sensitivity to various antibiotics, illustrates its adaptability and the challenges it poses in clinical management [2]. However, the rise in antibiotic resistance, notably to classes like carbapenems, cephalosporins, and fluoroquinolones, calls for rigorous antibiotic stewardship and susceptibility testing to inform effective treatment strategies [2].

In essence, Shewanella algae is emerging as a significant human pathogen, capable of causing severe infections and exhibiting notable antibiotic resistance. The uptick in infections, even in regions previously less affected, points to this pathogen's expanding influence and underscores the need for increased clinical vigilance. The case of the 88-year-old patient, characterized by severe symptoms and a complex clinical presentation, exemplifies the pathogen's potential for severity and the necessity for a comprehensive, multidisciplinary approach in its diagnosis, treatment, and prevention.

## Case presentation

**Figure 1:**
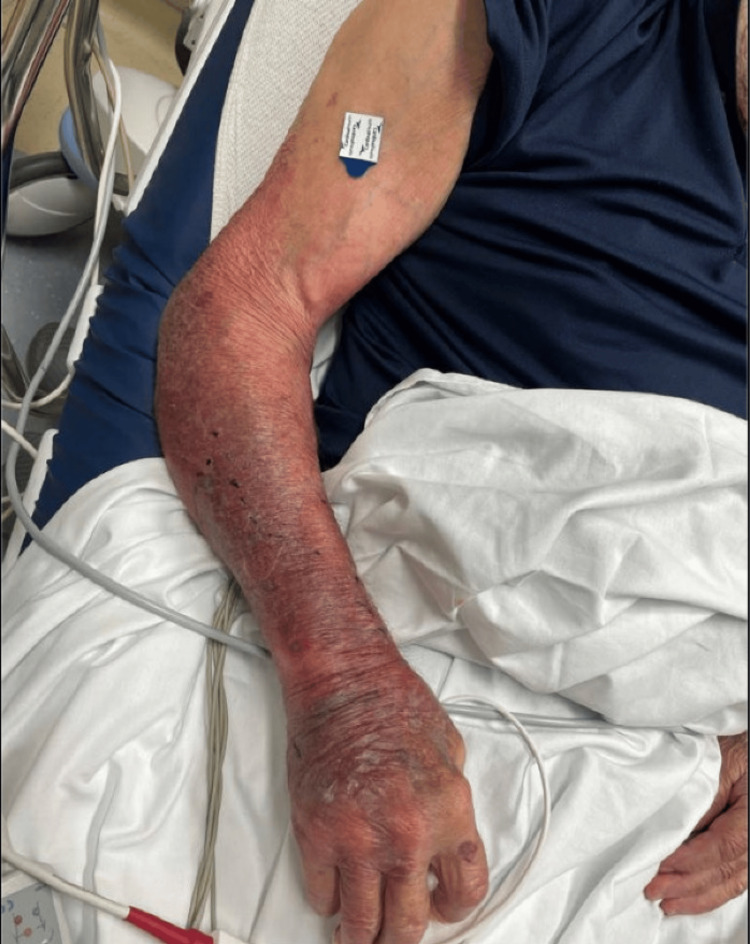
Erythema of the right forearm extending up to the mid upper arm.

An 88-year-old male, with a past medical history of mild cognitive impairment, lower extremity neuropathy, monoclonal gammopathy of unknown significance (MGUS), and benign prostatic hyperplasia (BPH), presented to the Emergency Room with worsening swelling and erythema of the right extremity and altered mental status. The patient was pinched by a crab at Davis Island Beach while he was crabbing on his dock with his grandchildren. The incident occurred when he reached into the crab trap to remove the crab. The patient sustained a small laceration to the dorsal ulnar side of his right hand, with resultant exposure to Tampa Bay water. The patient presented with rapidly progressing erythema on the right forearm, extending towards the axilla, which developed significantly within one to two hours (Figure 1). Concurrently, he exhibited symptoms of delirium. Treatment commenced with one dose of intravenous Levofloxacin and Cephalexin; however, his condition deteriorated. In the emergency department, his maximum temperature spiked to 103.2°F, and his pulse rate increased to 104 beats per minute, prompting a sepsis alert. Blood cultures were subsequently collected. The patient was administered 1.75 grams of Vancomycin, 1 gram of Cefepime, and 100 mg of Doxycycline intravenously to cover potential Vibrio infection. Additionally, he received 650 mg of Tylenol and 1 liter of Lactated Ringer's solution to manage fever and hydration. Imaging studies, including x-rays, did not reveal any bony abnormalities.

The patient underwent a three-day hospital stay during which blood cultures were consistently drawn until discharge. Initial blood cultures taken on the first day of the patient's admission showed no growth to date. By the second day, an external blood culture obtained a day before the patient's admission at Tampa General Hospital (TGH) positively identified Shewanella algae, confirming the pathogen's role in the clinical syndrome. Furthermore, the patient's laboratory results reflected a changing infection status and systemic response. Initially, an elevated white blood cell (WBC) count of 10.59 x 10^3/µL signaled a potential infection, accompanied by mild anemia with hemoglobin at 12.0 g/dL and concerns for bleeding with a platelet (PLT) count of 151 x 10^3/µL. The patient's elevated bilirubin at 1.7 mg/dL suggested liver involvement. By the day of a discharge, a reduction in WBC to 8.13 x 10^3/µL hinted at resolving infection, while Bilirubin improved significantly to 0.9 mg/dL, reflecting better liver function. The patient's condition improved over time and after more than 72 hours without growth in TGH blood cultures, indicating no ongoing infection, the patient was transitioned to oral Levofloxacin at the time of discharge. The patient’s hospital course was also complicated by episodes of atrial fibrillation which were managed with rate control strategies.

## Discussion

This case of severe cellulitis caused by Shewanella algae in an elderly patient complicated by sepsis and delirium offers several noteworthy insights. Shewanella algae infections are typically associated with milder clinical manifestations and are associated with conditions like malignancy, diabetes, and HIV [2,3]. This case, however, illustrates a more severe manifestation, including sepsis, delirium, and atrial fibrillation following saltwater exposure. This contrasts with the existing literature, which often portrays these infections as less severe. For instance, studies mostly describe mild to moderate presentations of Shewanella infections, reinforcing the uniqueness of this case's severity [4].

Shewanella algae is less commonly encountered in the U.S., with higher prevalence in warmer coastal regions [5]. Shewanella has been found to inhabit environments such as the Columbia River estuary and cause rare infections in military personnel and cases of necrotizing fasciitis and osteomyelitis [6-14]. The rarity of such infections in West Florida adds to the case's significance, as it highlights a need for increased awareness among healthcare professionals in regions where Shewanella is not commonly expected. The elderly patient in this case had multiple comorbidities, which likely contributed to the severity of the infection and its complications. This aspect of the case reinforces the importance of considering patient background in the management of infections. Previous literature supports the notion that comorbidities can significantly alter the clinical course of infections, making this case a valuable addition to the understanding of Shewanella infections in patients with complex medical histories [15].

The successful outcome in this case was largely due to prompt diagnosis and the initiation of an appropriate antibiotic regimen. This aligns with research that emphasizes the importance of early identification and treatment of rare infections like those caused by Shewanella [16]. Furthermore, the case illustrates the effectiveness of a multidisciplinary approach, including the management of complications like delirium and atrial fibrillation, as recommended in the comprehensive care guidelines for elderly patients with infections [17].

## Conclusions

This case of severe cellulitis caused by Shewanella algae in an elderly patient, complicated by sepsis and delirium, offers several noteworthy insights. These insights contribute to the existing literature due to the unusual constellation of symptoms and the patient's multiple comorbidities. This case serves as a unique illustration of how a seemingly innocuous blue crab pinch, resulting in saltwater exposure, led to a severe soft tissue infection by Shewanella, triggering sepsis and delirium in a multi-morbid individual. This case adds to the existing literature by emphasizing the need for healthcare providers to consider Shewanella as a potential pathogen when confronted with saltwater exposure and atypical clinical symptoms. Additionally, this case illustrates how quality patient care can lead to positive treatment outcomes even in elderly, multi-morbid patients infected with Shewanella.
